# The TorRS two component system regulates expression of TMAO reductase in response to high hydrostatic pressure in *Vibrio fluvialis*

**DOI:** 10.3389/fmicb.2023.1291578

**Published:** 2023-11-07

**Authors:** Na Liu, Ting Jiang, Wen-Peng Cui, Xiao-Qing Qi, Xue-Gong Li, Yuan Lu, Long-Fei Wu, Wei-Jia Zhang

**Affiliations:** ^1^Laboratory of Deep-Sea Microbial Cell Biology, Institute of Deep-sea Science and Engineering, Chinese Academy of Sciences, Sanya, China; ^2^University of Chinese Academy of Sciences, Beijing, China; ^3^Institution of Deep-sea Life Sciences, IDSSE-BGI, Sanya, China; ^4^International Associated Laboratory of Evolution and Development of Magnetotactic Multicellular Organisms, CAS, Sanya, China; ^5^College of Information Science & Engineering, Ocean University of China, Qingdao, China; ^6^Aix Marseille University, CNRS, LCB, Marseille, France

**Keywords:** deep-sea, high hydrostatic pressure, adaptation, TMAO reduction, two-component system, TorRS

## Abstract

High hydrostatic pressure (HHP) regulated gene expression is one of the most commonly adopted strategies for microbial adaptation to the deep-sea environments. Previously we showed that the HHP-inducible trimethylamine N-oxide (TMAO) reductase improves the pressure tolerance of deep-sea strain *Vibrio fluvialis* QY27. Here, we investigated the molecular mechanism of HHP-responsive regulation of TMAO reductase TorA. By constructing *torR* and *torS* deletion mutants, we demonstrated that the two-component regulator TorR and sensor TorS are responsible for the HHP-responsive regulation of *torA*. Unlike known HHP-responsive regulatory system, the abundance of *torR* and *torS* was not affected by HHP. Complementation of the Δ*torS* mutant with TorS altered at conserved phosphorylation sites revealed that the three sites were indispensable for substrate-induced regulation, but only the histidine located in the alternative transmitter domain was involved in pressure-responsive regulation. Taken together, we demonstrated that the induction of TMAO reductase by HHP is mediated through the TorRS system and proposed a bifurcation of signal transduction in pressure-responsive regulation from the substrate-induction. This work provides novel knowledge of the pressure regulated gene expression and will promote the understanding of the microbial adaptation to the deep-sea HHP environment.

## 1. Introduction

High hydrostatic pressure (HHP) exerts severe effects on cellular processes and may lead to impaired cell division, abolished motility, reduced enzymatic activities and even fatality (Welch et al., 1993; Balny et al., 2002; Bartlett, 2002). Microorganisms inhabiting deep-sea have evolved diverse strategies to adapt to the HHP environment, such as increase the proportion of unsaturated fatty acids in membrane, alter the respiration pathways and synthesis of compatible piezolytes. The adjustment of biomolecule components and biological function was generally implemented through pressure-regulated gene expression, which has been pervasively observed in diverse taxa and numerous biological pathways (Fernandes et al., 2004; Vezzi et al., 2005; Amrani et al., 2014; Jian et al., 2015; Wang et al., 2021). However, limited studies concerning pressure-responsive regulator has been carried out to date, and the regulation mechanism remains mostly obscure.

Studies of deep-sea strain *Shewanella violacea* DSS12 discovered a HHP-inducible glutamine synthetase (encoded by *glnA*), whose expression was under the control of sigma54 factor and two-component system NtrBC (Nakasone et al., 1999, 2002). HHP increased the abundance of the enhancer-binding regulator NtrC and therefore upregulated the expression of *glnA* (Nakasone et al., 2002). The other well studied pressure-responsive regulator is the ToxRS system from *Photobacterium profumdum* SS9 (Campanaro et al., 2012). The ToxRS forms an inner membrane-located transcriptional factor complex that is widely distributed in members of the family Vibrionaceae and regulates expression of over two dozen genes, including the outer membrane porin OmpH and OmpL in the deep-sea strain (Campanaro et al., 2012). It was presumed that changing of pressure modifies composition and structure of the cytoplasmic membrane, which further changes the abundance and activity of the regulator ToxR and the expression of its regulon (Bartlett and Chi, 1994; Welch and Bartlett, 1998). Remarkably, in both cases, a two-component system is responsible for the HHP-responsive regulation, and the intracellular level of the regulator changes along with pressure.

Trimethylamine N-oxide (TMAO) is a tertiary amine oxide widespread in sea water. As an osmolyte and a piezolyte, TMAO favors for the adaptation of deep-sea organism to the low-temperature and high-pressure environment (Gibb and Hatton, 2004; Yancey et al., 2014). It also functions as an electron acceptor for anaerobic respiration in diverse heterotrophic marine bacteria (Barrett and Kwan, 1985). The reduction of TMAO to trimethylamine (TMA) is catalyzed by the TMAO reductase (Tor), which has been most extensively studied in *Escherichia coli*. Genes encoding periplasmic terminal reductase (*torA*), inner membrane c-type cytochrome (*torC*) and a chaperone protein (*torD*) form the *torCAD* cluster, whose expression was induced by the presence of substrate through the periplasmic substrate-binding protein TorT and two-component regulatory system TorRS (Barrett and Kwan, 1985; Simon et al., 1994, 1995; Jourlin et al., 1996). Binding of TMAO to TorT promotes its interaction with the periplasmic domain of inner membrane histidine kinase TorS, triggers conformational changes and a series of phosphorylation in TorS and TorR (Jourlin et al., 1996). The conserved histidine in the classical transmitter domain of TorS is first autophosphorylated, and the aspartic acid at the receiver domain and the histidine in the C-terminal alternative transmitter domain are phosphorylated sequentially. The phosphoryl group is then transferred to the conserved aspartate in the receiver domain of the response regulator TorR, which brings TorR higher binding affinity with the promoter region of *torCAD* operon and eventually activates the transcription of the TMAO reductase (Jourlin et al., 1996, 1997; Ansaldi et al., 2000; Baraquet et al., 2006).

The expression of TMAO reductase was long known to be strictly regulated by substrate. However, recent studies in several deep-sea bacterial strains demonstrated that TMAO reductase can be induced by elevated pressure in the absence of TMAO (Campanaro et al., 2005; Zhang et al., 2016; Yin et al., 2018). It was first discovered by comparative transcriptomic analysis in piezophilic bacteria *P. profundum* SS9. Strain SS9 encodes three sets of TMAO reductase, one of them (PBPRA1467-1468) exhibited higher expression level at 28 MPa compared to that at atmospheric pressure (Vezzi et al., 2005; Le Bihan et al., 2013). Similar phenomenon was observed in a closely related strain, deep-sea luminous bacterium *P. phosphoreum* ANT-2200. Within its genome, four loci coding for TMAO reductase system consisting of a canonical *torR-torCAD* gene cluster, two *torECA* gene clusters and a *torCA* gene cluster were identified. Tandem mass spectrometry analysis and enzymatic activity staining showed that the abundance of TorA1 (PPBDW_v2_I20752) significantly increased by HHP (Zhang et al., 2016). In our recent work, we identified an HHP-inducible TMAO reductase in deep-sea pressure-tolerant strain *Vibrio fluvialis* QY27. Strain *V. fluvialis* QY27 is pressure-tolerant and capable of growing at pressures up to 50 MPa. It encodes two sets of TMAO reductases (*torAC* and *torZY*), and the expression of *torAC* is significantly induced by both substrate and elevated pressure, while the other set had a constitutive expression at a low level. By constructing deletion mutant, we further demonstrated that the HHP-inducible *torA* led to higher TMAO reduction efficiency under HHP condition, and improved pressure tolerance of strain QY27 in the presence of TMAO (Yin et al., 2018, 2019). Despite broad distribution in several deep-sea strains and an important role in bacterial adaptation to the HHP, the regulation mechanism of TMAO reductase in response to HHP remains unknown.

In this study, we identified the regulatory machinery responsible for the HHP-dependent induction of TMAO reductase in deep-sea strain QY27, and investigated its regulation mechanism in response to elevated pressure. By constructing mutants of the TorRS two-component system, we demonstrated that the regulator TorR is involved in both substrate- and HHP- responsive regulation of TMAO reductase in deep-sea strain QY27. Unlike the known HHP-responsive regulatory systems NtrBC and ToxRS, the expression level of *torR* and *torS* was not affected by HHP. Then we focused at the upstream sensor TorS. The complementation assay with *torS* carrying mutations at the conserved phosphorylation sites in *ΔtorS* knockout mutant showed that, the HHP-responsive phosphorylation path was different from the well-studied signal transduction pathway in substrate-dependent regulation, and only one of the three substrate-related phosphorylation sites is required for HHP-responsive regulation. Our results illustrated the TorS-mediate HHP-responsive regulation and suggested a novel regulation mechanism of TorRS regulatory system at the level of signal transmission.

## 2. Materials and methods

### 2.1. Bacterial strains and culture conditions

*Vibrio fluvialis* QY27 was cultured in YPG medium at 25°C as reported before (Yin et al., 2018). The cultivations of strain QY27 were carried out in syringes of 2.5 mL volume. The syringes were placed in high-pressure vessels (Feiyu Science and Technology Exploitation Co., Ltd., Nantong, China), and the hydrostatic pressure was applied with a water pump (Top Industrie, France) as described previously (Li et al., 2018). TMAO was supplemented to a final concentration of 1% (w/v) when needed. QY27 strains carrying pBBR1MCS2 derived plasmids were cultured in YPG medium supplemented with 30 μg/mL kanamycin. The biomass of QY27 cultures were measured with the absorption at 600 nm. The *E. coli* strains were cultured at 37°C with shaking. The diaminopimelic acid (DAP) auxotrophic *E. coli* strain WM3064 was cultured with the supplementation of 0.3 M of DAP. The *E. coli* strains carrying pBBR1MCS2 derived and pRE112 derived plasmids were maintained with 30 μg/mL kanamycin and 30 μg/mL chloramphenicol, respectively.

### 2.2. Construction of *torR* and *torS* deletion mutant and complementary strains

TorR deletion mutant was constructed using CRISPR-Cas9 system as previously described (Chen et al., 2018). The knockout plasmid pBBR-tac-Cas9-*torR* contained *cas*9 gene under the control of Ptac, the sgRNA targeting at *torR* and the 1 kb fragments flanking the *torR* gene as the template sequence for genome editing. The gRNA sequences for *torR* was 5’-AGTTGATGTCCAGCATCACC-3′. The primers used to amplify upstream and downstream fragments flanking *torR* were as follows: primer 1, 5’-TCCCCCCGGGGTTTGCGGCCAATATAGTAGTC-3′; primer 2, 5’-CGGGATCCACACCACTTTATGCTCATTC-3′; primer 3, 5’-CGGGATCCGCGTTGACGCAGAAAG-3′; and primer 4, 5’-TCCCCGCGGATTCTGTCATAGGAATGGC-3′. The plasmid pBBR-tac-Cas9-*torR* was synthesized by Suzhou Jinweizhi Biotechnology Co., Ltd. The plasmid was first transferred into the nutritional deficiency strain *E. coli* MW3064 by electroporation (voltage 1200 V, time 5.5 ms), and then introduced into strain QY27 by conjugation. The transformants were cultivated for 24 h with the presence of 0.2 mM isopropyl β-D-thiogalactoside (IPTG) that induced the expression of Cas9 protein for DNA cleavage.

The deletion mutant of *torS* was constructed by means of homologous recombination double exchange. To construct plasmid pRE112-*torS*, DNA fragments flanking *torS* were amplified from the genomic DNA of QY27 with primer 1, 5′- CATGCCATGGTACCCGGGATGCCTTCGTATTG-3′; primer 2, 5′- GAGCAGGCGAGGATCCAGGCTCAGC-3′; primer 3, 5′- CTGCTGAGCCTGGATCCTCGCCTGC-3′; and primer 4, 5′- CGATCCACTAGTTCTAGAGGGTGAGTTTGGCG-3′. The gentamycin gene cassette was acquired from the vector pUCGm. The three fragments were assembled by fusion PCR and introduced into pRE112. The resulting plasmid was transferred into the nutritional deficiency strain *E. coli* MW3064 by electroporation (voltage 1200 V, time 5.5 ms), and then introduced into strain QY27 by conjugation. The double crossover transformants were screened by a two-step strategy, first culturing on the plates of YPG with chloramphenicol and then on plates of YPG supplemented with 15% (w/v) sucrose.

The point mutated *torS* genes were synthesized by Suzhou Jinweizhi Biotechnology Co., Ltd. and introduced into pBBR1MCS2. The plamids carrying mutated *torS* were introduced into deletion mutant of *torS* by conjugation and screened by growth on YPG plates with kanamycin.

To carry out bi-parental conjugation, the donor strain of WM3064 cell carrying desired plasmid was cultivated at 37°C in LB medium to exponential phase. One milliliter of WM3064 culture was washed twice with LB medium before mixed with 1 mL of culture of QY27 strain. The mixture was suspended with 30 μL of YPG medium supplemented with DAP and placed on a filter on a plate of YPG medium with DAP overnight for conjugation. The mixture was transferred into an EP tube with YPG medium and incubated at 25°C for an hour before spread on plates for the selection of transformants. The full-length deletion of *torR* or *torS* gene and the complementation of *torS* from the plasmid were confirmed by PCR amplification and sequencing (Sangon biotech, China).

### 2.3. RNA extraction and real-time RT-PCR

Total RNA of QY27 cells was extracted as previously described (Yin et al., 2018). Briefly, approximately 10^7^ cells were collected and treated with trizol and chloroform. The nucleic acids were precipitated and washed with isopropanol and ethanol, and then dissolved in RNase-free water. Residual DNA was digested with DNase I and the reverse transcription was performed with a PrimeScript™II 1^st^ strand cDNA synthesis kit (TAKARA, Shiga, Japan). RT-PCR was conducted with StepOne Software (ABI). The relative expression of the target gene was normalized to the reference gene of *rpoD*. Three replications were set for each assay for the calculation of the mean value and the standard deviation.

### 2.4. Quantification of TMAO

TMAO was quantified by Raman spectroscopy analyses. The cultures were centrifuged at 12,000 rpm for 10 min to remove the cells, and the supernatants were preserved at −20°C before analyzed. The Raman spectra were collected with an acquisition time of 10 s in the range from 0 cm^−1^ to 2000 cm^−1^, using a portable fiber Raman system composed of a fiber optic Raman sensor RL-RP-785-(F/S)S (Beijing RealLight Technology Co., Ltd) and a spectrometer (ocean optics QEPro, ocean Insight, Shanghai). The peak at 751 cm^−1^ represents TMAO (P_TMAO_) and the peak at 2500 cm^−1^ represents H_2_O (P_H2O_). The peak area ratio was calculated by dividing the area value of P_TMAO_ with that of P_H2O_. To quantify concentration of TMAO, a standard curve was first established. The Raman spectra of YPG medium supplemented with different concentrations of TMAO (0, 0.2, 0.4, 0.6, 0.8, 1.0, and 1.2%) were collected and the area of P_TMAO_ and P_H2O_ peaks were measured and the ratio was calculated. For each sample, five spectra were collected, and the ratios of P_TMAO_/P_H2O_ were calculated for each spectrum and the average and standard deviation were calculated. The spectrum data were processed by NGSLabSpec and Origin softwares as previously described (Yin et al., 2018).

### 2.5. Preparation of TorR antibody and western-blot

The *torR* was cloned into pET-28(a) for the production of his-tagged TorR. TorR was expressed in *E. coli* BL21 (TAKARA, Shiga, Japan) and purified with Ni- affinity chromatography. Briefly, IPTG was added to a final concentration of 0.8 mM for induction. After 4 h’ induction at 28°C, cells were collected by centrifuge and washed twice with 20 mM Tris–HCl pH 8.0. The cell pellet was suspended in lysis buffer (10 mM imidazole, 20 mM Tris–HCl, 500 mM NaCl) supplemented with Pierce™ protease inhibitor (Thermofisher) and lysed by sonication. The cellular lysis was centrifuged at 5000 rpm for 30 min at 4°C, and the supernatant was then loaded to Ni-NTA Superflow (Thermo Scientific, Shanghai, China). The TorR was eluted with elution buffer containing 100 mM imidazole. The eluted fractions were analyzed by SDS-PAGE.

The antibody against TorR was prepared by ChinaPeptides (QYAOBIO) (Suzhou, China) and then purified with *E. coli* cells. For short, *E. coli* BL21 cells containing pET-28(a) plasmid were collected and washed with Tris–HCl buffer before incubated in pre-cooled acetone. The treated *E. coli* cells were air-dried into powder and incubated with the antiserum for the purification of TorR antibody.

For western-blot analysis, cells grown to exponential phase were collected and suspended in 40 mM Trhis-HCl (pH 7.4) containing Pierce™ protease inhibitor (Thermofisher) and lysed by sonication. The crude extract was collected by centrifuge and the protein concentration was determined by Pierce™ BCA Protein Assay Kits (Thermofisher). The crude extract was separated by SDS-PAGE and detected with anti-TorR as first antibody, HRP conjugated goat anti rabbit IgG as second antibody and developed with DAB horseradish peroxidase color development kit (Beyotime).

## 3. Results

### 3.1. Growth feature and expression of TMAO reductase under different cultural conditions

To better understand the regulation of TMAO reductase TorA in response to pressure, we first examined the effect of HHP on expression of *torA* at different growth phases. Strain QY27 was usually cultivated in half filled syringe (2.5 mL syringe filled with 1 mL medium and 1.5 mL air), to provide oxygen for respiration. However, recent studies showed that unlike most anaerobic respiratory systems, TMAO reductase is expressed under aerobic conditions, but with strong cell-to-cell fluctuation (Ansaldi et al., 2007; Roggiani and Goulian, 2015; Carey et al., 2018). Considering the consumption of oxygen will lead to a transition from aerobiosis to anaerobiosis, and complicate the regulation of TMAO reductase, we compared the cultures in half filled syringes (designated as aerobic condition) and fully filled syringes (designated as microaerobic condition).

**Figure 1:**
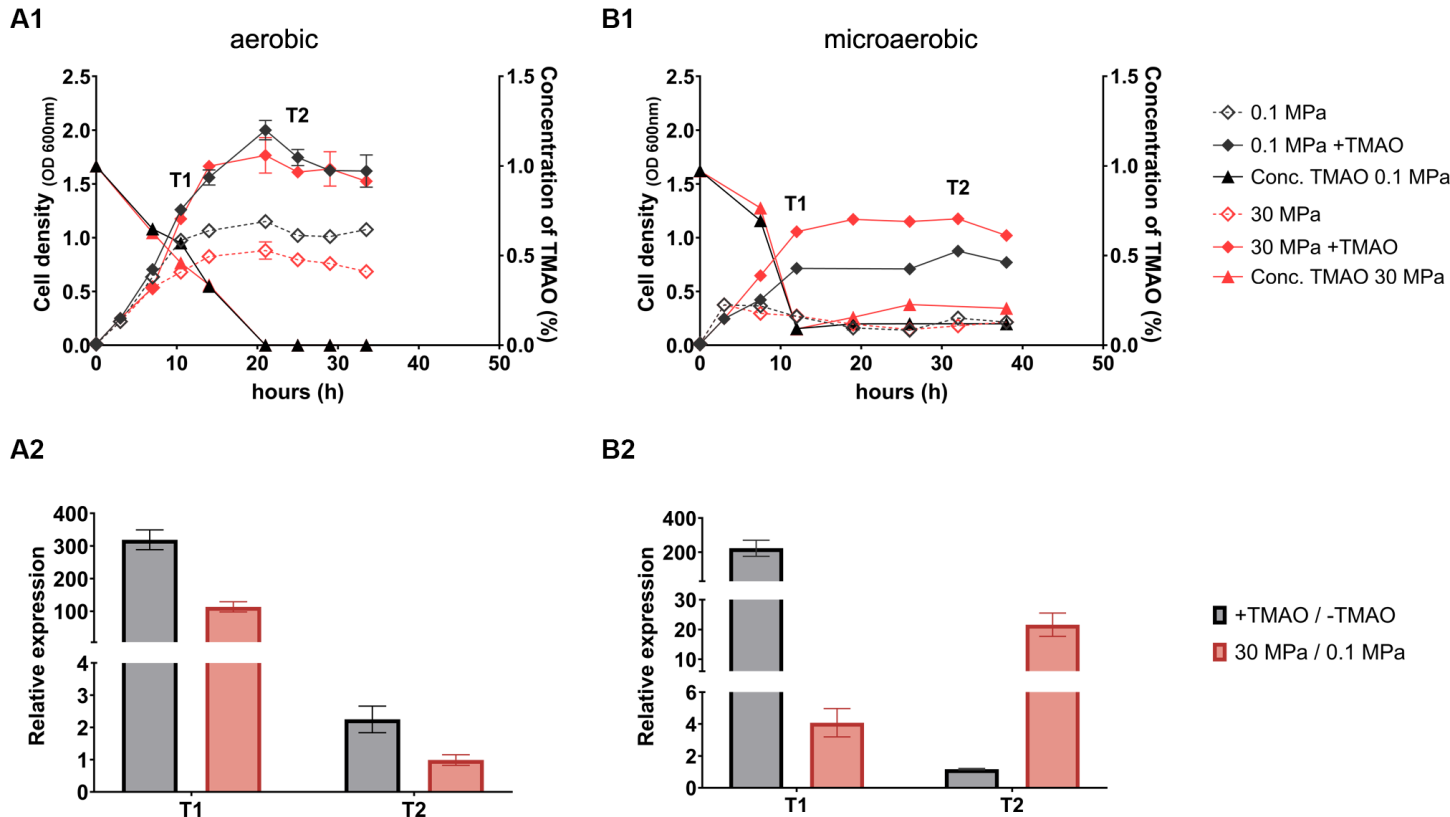
The growth, TMAO consumption and expression of TMAO reductase of QY27 under microaerobic and aerobic conditions. Panel **(A1,B1)** show the growth (curves with diamonds) and TMAO concentration (curves with triangles) of QY27 grown under aerobic and microaerobic condition, respectively. The lines in black represent cultures at atmospheric pressure (0.1 MPa) and lines in red represent cultures at high pressure (30 MPa) conditions. The dash lines represent cultures in plain medium and solid lines represent cultures with addition of TMAO. Panel **(A2,B2)** show the relative expression level of TMAO reductase catalytic subunit (*torA*) in aerobic and microaerobic cultures, respectively. The grey bars represent relative expression of *torA* in the presence of TMAO versus plain medium at 0.1 MPa, and the red bars represent relative expression of *torA* at 30 MPa versus 0.1 MPa in plain YPG medium. The T1 and T2 indicate two sampling points as marked in the panel **A1** and **B1**. The average values and standard deviations are resulted from three replicates.

The growth of strain QY27 under aerobic condition was consistent with our previous observation. The culture entered stationary phase after around 15 h’ cultivation in plain YPG medium, with cell densities of 1.0 OD_600 nm_ at 0.1 MPa and 0.8 OD_600 nm_ at 30 MPa, respectively. Addition of TMAO extended the exponential phase, and cultures at both pressures entered stationary phase with cell densities of approximately 1.7 OD_600 nm_ at approximately 20 h, when TMAO was exhausted (Figure 1A1). The qPCR analyses were performed to examine expression of TMAO reductase at two growth phases: cells grown to exponential phase with maximum TMAO consumption rate (T1), and cells grown to stationary phase when TMAO was undetectable (T2). The results showed that substrate and HHP induced the expression of *torA* in cells at exponential phase by around 300-fold and 100-fold, respectively, while no induction was observed in cells grown to stationary phase (Figure 1A2).

When grown under microaerobic condition, cultures in plain medium had a very short exponential phase and entered stationary phase in 4 h, with maximum cell-density of around 0.4 OD_600 nm_. Addition of TMAO increased cell densities to around 0.8 and 1.2 OD_600 nm_ at 0.1 MPa and 30 MPa, respectively (Figure 1B1). Comparing to cultures at aerobic condition, the consumption of TMAO was faster under microaerobic condition (12 h under microaerobic condition versus 20 h under aerobic condition). The substrate-dependent induction of *torA* was observed in cells at exponential phase alone (around 200-fold) while HHP-dependent induction was observed in cells collected at both exponential phase (around 4-fold) and stationary phase (around 20-fold) (Figure 1B2). Collectively, elevated pressure up-regulated *torA* under both aerobic and microaerobic conditions, but the degree and time window of induction varied. To minimize the interference from oxygen and focus on the HHP-dependent regulation of TMAO reductase, microaerobic cultural condition was used in this study.

### 3.2. The HHP-responsive induction of *torA* requires two-component system TorRS

**Figure 2:**
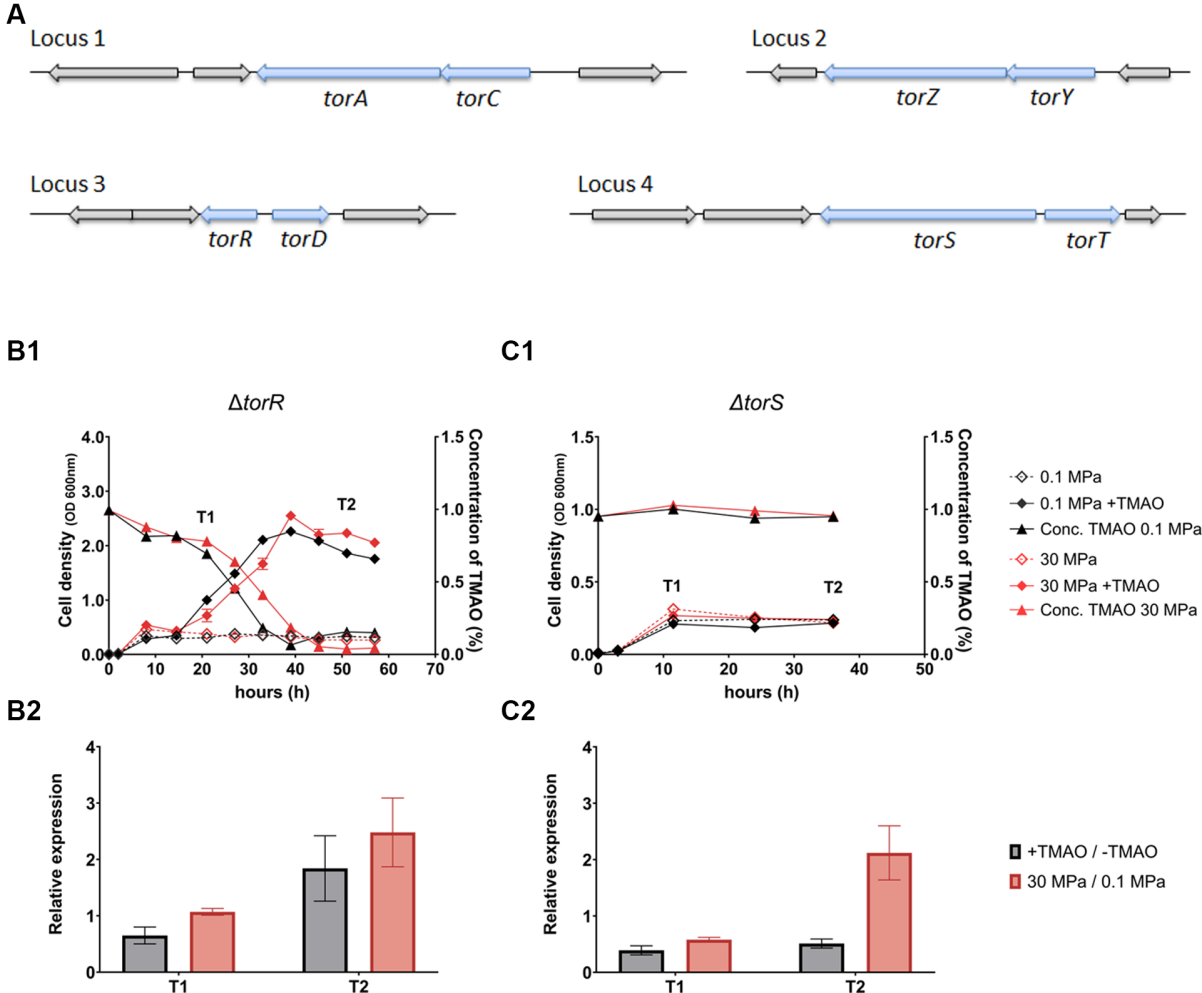
The organization of genes involved in synthesis of TMAO reducase, and growth, TMAO consumption and expression of TMAO reductase of Δ*torR* and Δ*torS* mutants. Panel **(A)** shows the four loci containing genes involved in synthesis of TMAO reductase (arrows in blue) in the genome of strain QY27. Panel **(B1,C1)** show the growth (curves with diamonds) and TMAO concentration (curves with triangles) of Δ*torR* and Δ*torS* mutants, respectively. The lines in black represent cultures at atmospheric pressure (0.1 MPa) and lines in red represent cultures at high pressure (30 MPa) conditions. The dash lines represent cultures in plain medium and solid lines represent cultures with addition of TMAO. Panel **(B2,C2)** show the relative expression level of TMAO reductase catalytic subunit (*torA*) in Δ*torR* and Δ*torS* mutants, respectively. The grey bars represent relative expression of *torA* in the presence of TMAO versus plain medium at 0.1 MPa, and the red bars represent relative expression of *torA* at 30 MPa versus 0.1 MPa in plain YPG medium. The T1 and T2 indicate sampling times as marked in the panel **B1** and **C1**. The average values and standard deviations are resulted from three replicates.

Examination of QY27 genome identified eight components involved in the synthesis of TMAO reductase: two sets of TMAO reductase catalytic subunit (TorA and TorZ) and corresponding cytochrome c (TorC and TorY), a TMAO reductase maturation chaperon TorD, a periplasmic substrate TMAO binding protein TorT, and the substrate-responsive two-component regulatory system composed of a histidine kinase TorS and a response regulator TorR (Figure 2A). The genes were dispersed in four loci: including two operons consist of *torCA* and *torYZ*, respectively, the third consisted of *torS* and *torT*, and the fourth of *torR* and *torD*. It was noted that, although *torS* and *torR* are adjacent to *torT* and *torD*, respectively, and they transcribe in opposite directions. The *torS* and *torR* locate on the reverse strand, upstream of *torT* and *torD* that are both on the forward strand. Sequence analysis showed that TorS and TorR from strain QY27 shared merely 33 and 53% identity in amino acid sequences to their homologs in *E. coli*, respectively.

To examine whether the TorRS regulatory system engaged in not only substrate-dependent regulation, but also HHP-responsive regulation of *torA* in strain QY27, we constructed deletion mutants of *torR* and *torS*, respectively, and characterized their growth and expression of *torA*. The two mutants exhibited same growth curve as the wild-type strain when cultivated in plain medium, but reacted differently to the supplementation of TMAO, as the addition of TMAO slowly stimulated the growth of *ΔtorR* but not *ΔtorS* (Figures 2B1, C1). TMAO was utilized by the mutant *ΔtorR* under both pressure conditions, but less efficient compared to the wild-type strain. It took the *ΔtorR* around 40 h and 45 h to exhaust TMAO at 0.1 MPa and 30 MPa, respectively, which was over 3-times longer than the wild-type strain (approximately 12 h at both pressures) (Figure 2B1). In contrast, the TMAO consumption was completely abolished in *ΔtorS* deletion (Figure 2C1).

Despite differences in growth profile and TMAO utilization, similar expression pattern of *torA* was observed in the two mutants. As expected, *torA* was no longer induced by the presence of TMAO. Meanwhile, its expression was weakly up-regulated (barely 2-fold) by elevated pressure, and the induction appeared only at stationary phase (Figure 2B2, C2). The observation that mutation of *torR* or *torS* led to abolished substrate-dependent regulation and severely impaired HHP-responsive regulation suggested that the TorRS regulatory system is responsible for both the substrate- and HHP- responsive regulation of TMAO reductase in deep-sea strain QY27.

### 3.3. The expression of TorR and TorS are not affected by HHP

**Figure 3:**
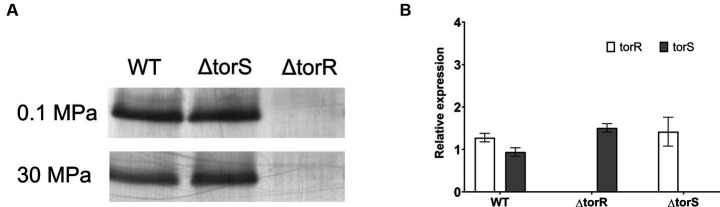
Effect of elevated pressure on the expression of TorR and TorS. Panel **(A)** shows the detection of TorR by western-blot in the wild-type (WT), Δ*torR* and Δ*torS* strains. Panel **(B)** shows the relative expression level of torR and torS under 30 MPa versus 0.1 MPa in plain YPG medium.

A common feature of the two known pressure-responsive regulatory systems is the altered abundance of regulator (NtrC and ToxR) by the function of pressure (Welch and Bartlett, 1998; Nakasone et al., 2002). To examine whether the TorRS system responds to elevated pressure through the same mechanism, we analyzed the abundances of regulator TorR and histidine kinase TorS in the wild-type strain and the two mutants. Western-blot and qPCR analyses showed comparable abundance of TorR and transcripts of *torR* in the wild-type QY27 strain and the Δ*torS* mutant strain grown at 0.1 MPa and 30 MPa (Figure 3). We circumvented the difficulty in purifying the integral membrane protein TorS to raise antibodies for western-blot by the qPCR analysis. The transcription of *torS* was apparently not affected by elevated pressure nor the deletion of *torR* **(**Figure 3**)**. Taken together, the constitutive expression of TorR and TorS under different pressures suggested that, unlike the known HHP-dependent regulation system NtrBC and ToxRS, TorRS system probably regulate gene expression in response to HHP through a distinct mechanism.

### 3.4. One conserved phosphorylation site in TorS alone is involved in the HHP-responsive regulation by TorRS

**Figure 4:**
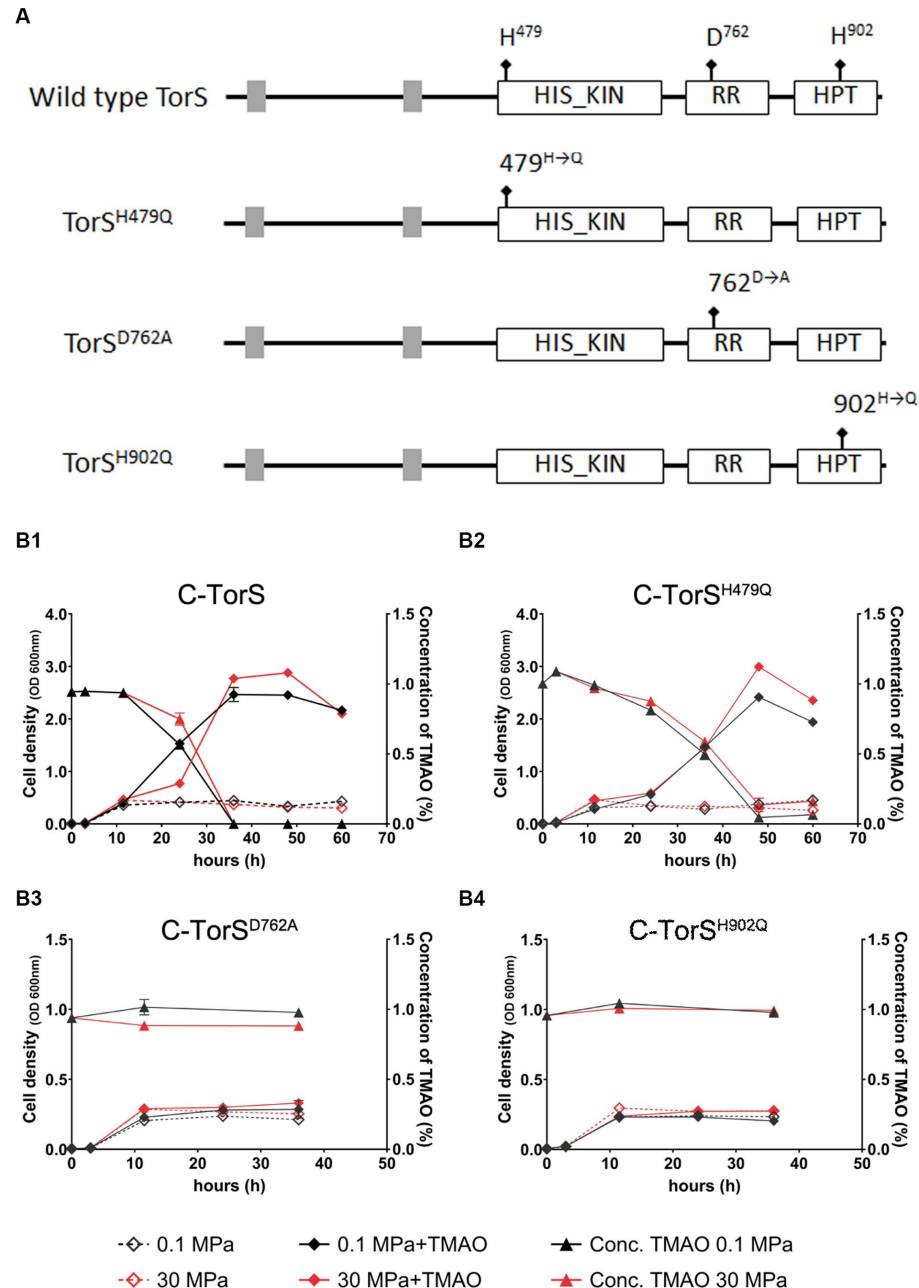
Schematic of the wild-type and mutated TorS and the growth and TMAO consumption of QY27 carrying wild-type and mutated TorS. Panel **(A)** shows the conserved domains and phosphorylation sites in wild-type TorS and mutated TorS. The grey boxes represent trans-membrane segments. The mutated phosphorylation sites are marked with arrows with square at the end. Abbreviations: HIS_KIN, histidine kinase domain; RR, response regulatory domain; HPT, histidine-containing phosphotransfer domain. Panel **(B1–B4)** show the growth (curves with solid squares) and TMAO concentration (curves with open squares) of complementary strains C-TorS, C-TorS^H479Q^, C-TorS^D762A^ and C-TorS^H902Q^, respectively. The black and red lines represent cultures in YPG medium at atmospheric pressure (0.1 MPa) and high pressure (30 MPa), respectively. The dash lines represent cultures in plain medium and solid lines represent cultures with addition of TMAO. The average values and standard deviations are resulted from three replicates.

The conveying of signal in a two-component regulatory system was realized through a series of phosphoryl-transfer reactions. Three amino acids in TorS are essential for the autophosphorylation and phosphoryl-transfer reaction during substrate-dependent regulation in *E. coli*. They are the two histidines in the classical transmitter domain (H^443^) and the alternative transmitter domain (H^850^), and an aspartic acid in the sensor receiver domain (D^723^) (Jourlin et al., 1996, 1997). Sequence analysis of TorS from QY27 recognized a conserved histidine kinase (HIS KIN) domain, a response regulatory (RR) domain and a histidine-containing phosphotransfer (HPT) domain, as well as three phosphorylation sites (H^479^, D^762^ and H^902^) (Figure 4A).

**Figure 5:**
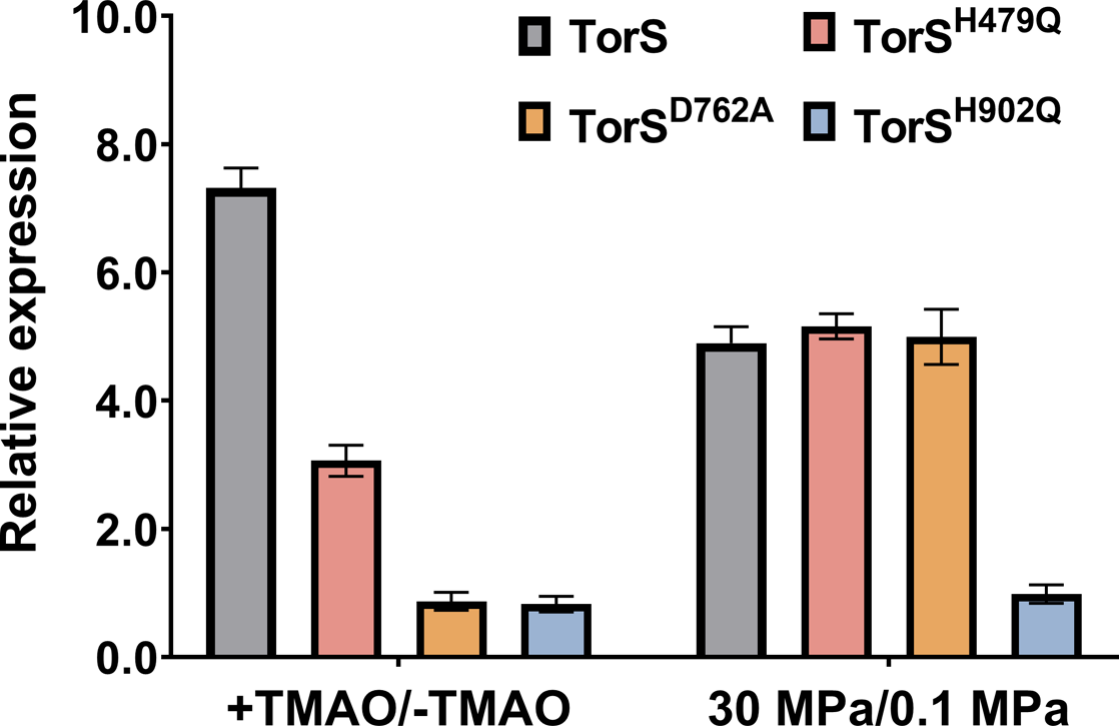
The effect of substrate and HHP on expression of QY27 carrying wild-type and mutated TorS. Bars present the relative expression level of torA in different strains. + TMAO/− TMAO represents expression with addition of TMAO in relative to that in plain medium, at 0.1 MPa; 30 MPa/0.1 MPa represents expression under 30 MPa in relative to that under 0.1 MPa, in plain medium.

To elucidate the mechanism of HHP-responsive regulation of TorRS, we replaced the two histidines with glutamines (TorS^H479Q^ and TorS^H902Q^), and the aspartic acid with alanine (TorS^D762A^), respectively, and introduce the mutated TorS proteins into the *ΔtorS* mutant (Figure 4A). Both growth curves and quantification of TMAO suggested that in trans expression of the wild-type TorS (C-TorS) restored TMAO reduction (Figure 4B1). Expression of mutated TorS^H479Q^ partially complemented the TMAO reduction. Strain C-TorS^H479Q^ was capable of reducing TMAO, but with a relatively lower efficiency. It exhausted the TMAO by around 50 h, much longer than the 35 h required by the complementary strain carrying wild-type TorS (Figure 4B2). Expression of the other two mutated TorS proteins (TorS^D762A^ and TorS^H902Q^) did not change the growth profile of the *ΔtorS*, thus failed to utilize TMAO (Figures 4B3,B4). The effect of TMAO on the expression of *torA* was in accordance with the growth phenotype. The presence of substrate up-regulated *torA* by approximately 7- and 3-fold in strain C-TorS and C-TorS^H479Q^, while a constitutive expression profile was observed in strains TorS^D762A^ and TorS^H902Q^ (Figure 5). Surprisingly, the expression of *torA* under HHP exhibited a different profile. It was up-regulated by approximately 5-folds in strains carrying wild-type TorS, TorS^H479Q^ and TorS^D762A^, while no induction by HHP was observed in strain C- TorS^H902Q^ (Figure 5).

**Figure 6:**
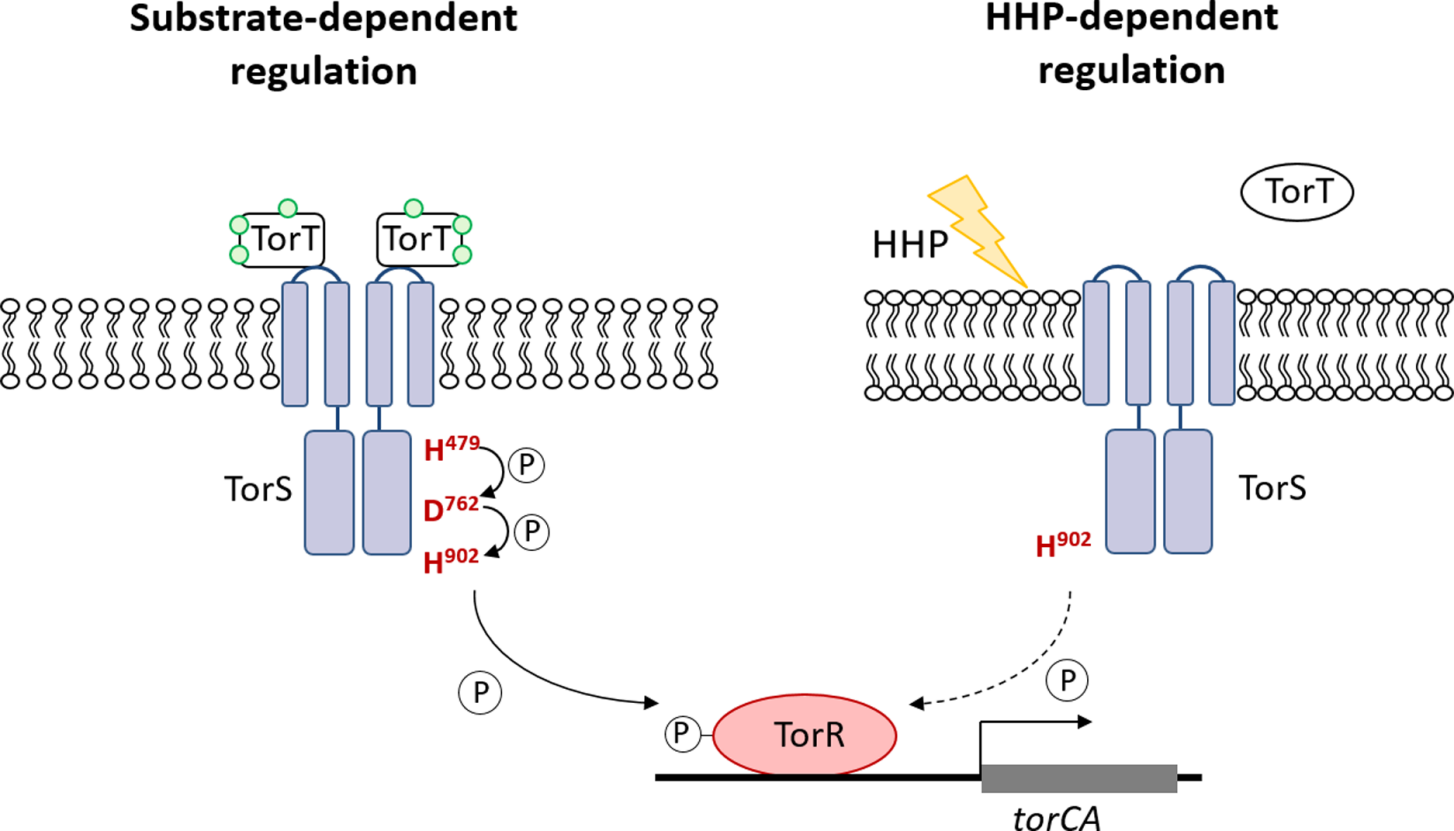
Diagram of substrate- and HHP-responsive regulation of TorRS system in deep-sea strain QY27. The diagram shows signal transduction pathway of TorRS system in stain QY27 under different conditions. Substrate triggered phosphoryl-transfer in strain QY27 involves all the three phosphorylation sites, but the histidine located in the alternative transmitter domain alone is required for the HHP-dependent regulation.

Taken together, the growth profiles and the qPCR results showed different involvement of the three conserved phosphorylation sites in TorS during substrate- and HHP-dependent regulation of *torA*. Mutation of the histidine in the classical transmitter domain (H479Q) of TorS partially impaired the substrate-dependent regulation but had no influence on the HHP-dependent regulation. The mutation of the aspartic acid in the sensor receiver domain (D762A) preserved only the function of HHP-responsive regulation, while substitution of the histidine in the alternative transmitter domain (H902Q) abolished the capacity to induce *torA* in response to both stimuli. Apparently, the TorRS regulatory system from deep-sea strain QY27 adopted different signaling pathways during substrate- and HHP- dependent regulation. Substrate triggered phosphoryl-transfer was same as the classical TorRS system from *E. coli*, with all the three conserved phosphorylation sites are involved. However, the phosphorylation site located in the alternative transmitter domain alone is essential for the HHP-dependent regulation (Figure 6).

## 4. Discussion

The expression of TMAO reductase was long believed to be strictly controlled by substrate, until the discovery of HHP induced expression of TMAO reductases in several deep-sea bacteria (Vezzi et al., 2005; Zhang et al., 2016; Yin et al., 2018). However, the regulator responsible for the pressure-dependent induction and its molecular mechanism remained unknown. In this study, we demonstrated that the TorRS two-component regulatory system was involved in the pressure-dependent induction of *torA*. To date, only two regulatory systems (NtrBC and ToxRS) are known responsible for HHP-dependent regulation. In both cases, the abundance of the regulator changes along with elevated pressure, and is thus believed a common strategy of pressure sensing (Bartlett and Chi, 1994; Nakasone et al., 1998, 1999, 2002). Surprisingly, abundances of both TorR and TorS were not affected by elevated pressure, while a distinct phosphorylation pathway different from substrate-responsive regulation was activated during HHP-responsive regulation.

Microorganisms receive diverse external stimuli, such as temperature and viscosity, by membrane and converts them into chemical signals through membrane embedded or associated protein complex. For example, inner membrane histidine kinase DesK detects low temperature through the increased thickness of membrane and activates the synthesis of unsaturated fatty acids which helps to maintain the fluidity of cellular membrane (Inda et al., 2014, 2016; Fernandez et al., 2019). HHP has same effect on cellular membrane as low temperature and was possibly sensed through similar mechanism (DeLong and Yayanos, 1985; Siebenaller and Garrett, 2002; Kawamoto et al., 2011). By addition of membrane-perturbing anesthetics, it has been demonstrated that the status of membrane played an important role in pressure sensing by ToxRS regulatory system (Welch and Bartlett, 1998). In the TorRS regulatory system, the sensor TorS is predicted to be anchored to the cytoplasmic membrane with two trans-membrane segments (12–34, and 334–355 residues) and thus possessing the structural basis to sense the changes in pressure through alteration of membrane. We noticed that truncated TorS lacking the first trans-membrane segment (TorS^35-958^) failed to regulate expression of *torA* in response to HHP (data not shown). It suggested the possibility that membrane localization is required for TorS to sense the stimulus of HHP. Mutation studies of residues within or close to the transmembrane segments may provide more biochemical evidence to illustrate the mechanism of HHP sensation by the TorRS regulatory system.

By constructing mutated TorS, we demonstrated that the HHP-responsive regulation of TorRS system was carried out through an alternative signaling pathway different from the well-known substrate-dependent regulation. All the three conserved phosphorylation sites in TorS were involved in the substrate-dependent regulation in deep-sea strain QY27, while the histidine located in the C-terminal alternative transmitter domain (H^902^) alone participated in the HHP-dependent regulation. It is generally believed that autophosphorylation of the histidine in the classical transmitter domain initiates signal transduction of two-component regulatory systems. Its absence in the HHP-responsive signaling pathway brings up a series of questions. For example, does the histidine located in the C-terminal alternative transmitter domain (H^902^) receive the phosphoryl group directly from ATP (autophosphorylation), or from other residues. It has been shown that the alternative transmitter domain of TorS from *E. coli* could be phosphorylated through a phosphotransfer involving a protein other than TorS (Jourlin et al., 1997). But the donner of the phosphoryl group to H^902^ of TorS in deep-sea strain QY27, and its downstream receiver remain unknown for now.

In addition, we showed that the TorA could be induced by HHP under both aerobic and microaerobic conditions, but the induction was more significant during exponential phase under aerobic condition, and during stationary phase under microaerobic condition. Since the pressure remained stable throughout the growth experiment, this difference suggested that the HHP-responsive regulation of TorA in deep-sea strain QY27 might be correlated with other physicochemical or physiological factors. Increasing evidence showed that several extreme environmental conditions, e.g., pressure, temperature, salinity, and pH, had similar effect on microbial cells and will interfere with the redox homeostasis. Accordingly, a common adaptation mechanism is adopted to cope with different types of stresses (Zhang et al., 2015; Wang et al., 2021; Li et al., 2023). Studies in *E. coli* showed that TorRS system also regulates genes involved in alkaline-resistance, such as *tnaA*, *gadA* and *hdeABD*, facilitating the cells to deal with the increase of pH resulted from TMAO reduction (Bordi et al., 2003). It would be of interest to identify the regulon of TorRS in response to change of pressure and their involvement in the adaption to the HHP environments.

In this study, we noticed that despite a constitutive expression of *torA* in both *ΔtorR* and *ΔtorS* mutants, the former was still capable of TMAO reduction slowly, but not the latter. It suggested the existence of another TMAO reductase whose function required the presence of TorS but not TorR. Previously, we have showed that, between the two TMAO reductase systems harbored by strain QY27, the *torCA* played a dominant role and was inducible by HHP, while the *torZY* expressed at a much lower level and its function and regulation were still mystery (Yin et al., 2018). It is plausible that the reduction of TMAO observed in mutant *ΔtorR* was catalyzed by TorZY. Further investigation will be necessary to decipher the function of TorS and if there is another regulator that replaces TorR in the regulation of TorZY. Collectively, we identified that TorRS two-component system is responsible for the HHP-responsive regulation of TMAO reductase and discovered that HHP triggered a novel signal transduction pathway of TorRS system that was different from the classical substrate-dependent regulation. These findings provide novel understanding of the regulation of energy metabolism in response to HHP and the microbial adaptation to the deep-sea environment.

## Data availability statement

The raw data supporting the conclusions of this article will be made available by the authors, without undue reservation.

## Author contributions

NL: Conceptualization, Formal analysis, Investigation, Methodology, Writing – review & editing. TJ: Conceptualization, Formal analysis, Investigation, Methodology, Writing – review & editing. W-PC: Methodology, Writing – review & editing. X-QQ: Investigation, Project administration, Writing – review & editing. X-GL: Formal analysis, Supervision, Writing – review & editing. YL: Methodology, Supervision, Writing – review & editing. L-FW: Conceptualization, Funding acquisition, Supervision, Writing – review & editing, Writing – original draft. W-JZ: Conceptualization, Funding acquisition, Supervision, Writing – original draft, Writing – review & editing.
